# Digital Light Processing Resins with Programmable Shape Memory for Biomedical Applications

**DOI:** 10.1021/acs.biomac.3c01276

**Published:** 2024-07-29

**Authors:** Ana A. Aldana, Tobias Kuhnt, Ramiro Marroquin Garcia, Lorenzo Moroni, Matthew B. Baker

**Affiliations:** † Department of Complex Tissue Regeneration, MERLN Institute for Technology-Inspired Regenerative Medicine, Maastricht University, P.O. Box 616, Maastricht 6200 MD, The Netherlands; ‡ Department of Instructive Biomaterials Engineering, MERLN Institute for Technology-Inspired Regenerative Medicine, Maastricht University, P.O. Box 616, Maastricht 6200 MD, The Netherlands

## Abstract

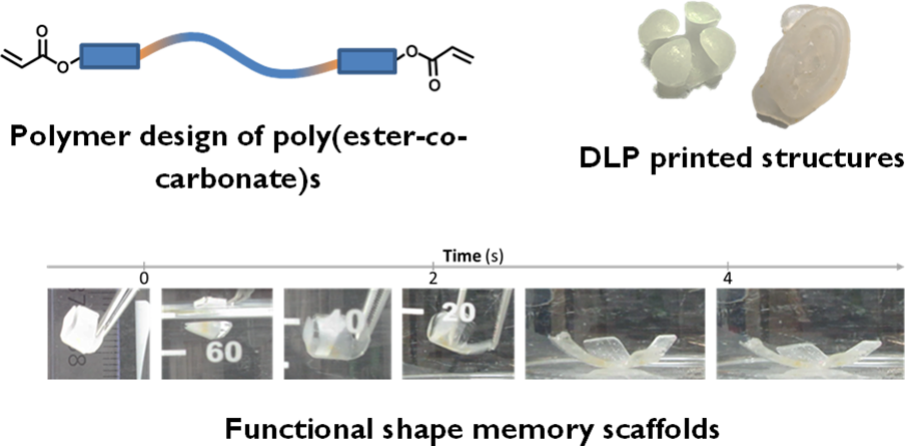

The creation of biodegradable and biocompatible shape memory polymers amenable to biofabrication techniques remains a challenge. The ability to create shape-changing biodegradable objects that are triggered at body temperature opens up possibilities in tissue engineering, minimally invasive surgery, and actuating bioimplants. Merging Digital Light Processing (DLP) printing with shape memory polymers brings us closer to new smart biomedical outcomes. Previously, we developed a poly(caprolactone-*co*-trimethylenecarbonate) urethane acrylate resin for the DLP fabrication of biodegradable 3D objects. In further studies, we observed that some of these resins possessed shape memory properties, triggered by body temperature (37 °C). In this subsequent study, we explored the shape memory properties and tunability of this resin family via changes in copolymer composition, molecular weight, and identity of the acrylate end-capping unit. We found that we could create a library of shape memory resins, amenable to DLP printing, which allowed the creation of shape-actuating structures with some tunability over the speed of shape memory and mechanical properties. We observed that increased mole fraction of caprolactone in the copolymer and increased molecular weight of the polymer led to a decrease in speed of the shape memory switch. Furthermore, we observed a trade-off between the composition and the end-capping moiety on the mechanical properties of the polymers. These polymeric resins were able to be processed into shapes that were able to perform work, including the release of cargo and grabbing/lifting of an object. This platform now provides a way to tune the speed and mechanical properties of a shape memory DLP object created from common and scalable polymerization techniques. This work ultimately provides a new platform to develop customizable and biodegradable devices capable of actuating and delivery devices for numerous biomedical applications.

## 1 Introduction

The ability to create biocompatible 3D scaffolds with temporal properties using a stimulus to trigger shape changes opens up new possibilities for designing smart biomedical devices. While shape changes of biomedical devices are mainly limited to swelling/shrinking (volume increase/decrease), the use of shape memory materials (SMMs) brings the opportunity to transform completely the material’s shape.^1−3^ This important class of stimuli-responsive materials, with the capability to change the shape in response to an external stimulus, have a great potential for biomedical applications,^4^ such as tissue-engineered scaffolds, cardiovascular grafts, stents, and drug delivery systems, among others.

Shape memory polymers (SMPs) represent the most useful class of SMMs for biomedical applications owing to material properties (e.g., mechanics and hydrophilicity) closer to native tissues and their biocompatibility. A small device made of SMPs can be inserted into the body and then adopt its larger functional shape, facilitating minimally invasive procedures. Furthermore, biodegradable and bioresorbable SMPs can also prevent a second surgery for removing implanted materials^2^ or support tissue formation and integration.

SMPs have gained even more attention since 4D printing emerged in the past decade.^5−8^ By merging additive manufacturing with SMPs, we are now able to fabricate complex stimuli-responsive structures with programmable morphing changes. There have been several notable advances in this area within the past few years. For example, copolymers of poly(glycerol sebacate)^9,10^ and poly(glycerol dodecandioate)^11,12^ as well as several resins based on derivatization of natural oils like soybean,^13^ among other vegetable oils,^14^ have all shown promise in the 3D fabrication of shape memory objects. Among the additive manufacturing techniques, light-based biofabrication like Digital Light Processing (DLP) results in high resolution and fast printing times.^17,18^ DLP has been recently explored in regenerative medicine owing to its high accuracy to print complex scaffolds like vascular branches. More traditionally explored synthetic polymers like poly(propylene fumarate) star polymers^15^ and poly(lactide-*co*-caprolactone)^16^ resins have shown significant progress in creation of shape memory objects for biomedical applications by using light-based fabrication techniques like DLP. However, the lack of customizable biodegradable and biocompatible resins made of shape memory polymers still remains a significant bottleneck.

Recently, we have designed new resin formulations based on copolymers synthesized with the well-known monomers caprolactone (CL) and trimethylenecarbonate (TMC).^19,20^ Inspired by work with poly(lactide-*co*-trimethylene carbonate),^21^ we wanted to create less crystalline and slower degrading objects that could be amenable to 3D printing via DLP. The DLP-printed scaffolds showed tunable mechanical properties, degradation, and attached human mesenchymal stromal cell morphologies by controlling the monomer ratio within the copolymer. Among the investigated formulations, we found that the printed scaffolds made of the copolymer with high CL percentage (75 and 90) exhibited thermal transitions (melting and crystallization) that may enable a shape memory effect owing to the CL crystalline domain formation.

The shape memory properties of polycaprolactone (PCL), together with its biocompatibility and biodegradability, have motivated an increasing number of researchers to explore the macromolecular design for modulating the shape recovery (time and temperature, which trigger the response).^22−26^ Notably, here, we use the term biodegradability, although PCL has been shown to be capable of both enzymatic biodegradation and hydrolytic degradation.^27^ Most of the approaches include covalently cross-linked low molecular weight PCL, where the cross-links are indispensable to endow the material with a shape memory effect. Tian et al. have developed PCL networks using thiol–ene chemistry for covalent cross-linking.^24^ PCL with several molecular weights (from 4000 to 10000 kg/mol) was end-capped with acrylate groups to then react with a tetrathiol cross-linker. This approach showed that the melting transition temperature could be tuned by varying the molecular weight of PCL, which consequently affects crystallinity, mechanical properties, and shape memory performance. Not only can the molecular weight modulate the shape memory properties of these polymers but also it has been shown that cross-link functionality, spatial distribution, and the presence of pendant groups or comonomers within the polymer chain can have a large effect on the overall shape memory performance.

While significant research had been performed on PCL-based networks for shape memory materials, there are only a few studies exploring these materials for DLP technology. Invernizzi et al. have designed a PCL-based resin, which was combined with 2-ureido-4-[1*H*]-pyrridinone (UPy) for bringing self-healing properties to the material.^6^ The printed materials kept both self-healing and shape memory properties, where the melting transition temperature was 55 °C. However, merging the printability with the shape memory behavior triggered relevant physiological conditions (around 37 °C), which is the main challenge to overcome for biomedical applications. Furthermore, while many shape memory polymer platforms show simply a change in shape, the ability of the object to perform useful work is also interesting for a variety of fields within (drug delivery and minimally invasive surgery) and outside (soft robotics and responsive textiles) biomedical engineering. Currently, the ability of 3D-printed objects from shape memory polymers to perform useful tasks is also a challenge.

Herein, we explore the ability to tune the shape memory effect of these polymers by rationally designing macromolecular architecture, their tunability of shape memory based on the structure, their ability to form 3D-printed objects, and the amenability to make these 3D biocompatible structures produce work (delivery and gripping).

## 2 Experimental Section

### 2.1 Materials

l-Lysine diisocyanate ethyl ester (>96%, LDI) was obtained from Actu-All Chemicals, trimethylene carbonate (99%, TMC) was provided by Huizhou Foryou Medical Devices, ε-caprolactone (99%, CL) and 2-hyrdroxyethyl acrylate^28^ were obtained from Alfa Aesar, diphenyl (2,4,6-trimethylbezoyl) phosphine oxide (TPO) and calcium hydride (CaH_2_) were obtained from Sigma-Aldrich, tetrahydrofuran (anhydrous, ≥99%, THF), tin^22^ 2-ethylhexanoate (92.5%, Sn(II)Oct), methanol (99.5%), triethylene glycol (≥99%, TEG), and toluene (≥99.8%, anhydrous) were obtained from Merck, 2-(2-ethoxy-ethoxy) ethyl acrylate (EOEOEA) was provided by TCI Chemicals, and chloroform (98%, CHCl_3_) was obtained from VWR. CL was dried for 12 h over calcium hydride and distilled under reduced pressure. TMC was lyophilized for 48 h before polymerization, and Sn(II)Oct was distilled under reduced pressure before use. All other chemicals were used as received.

### 2.2 Synthesis of PCTAc

The general procedure for photopolymerizable copolymer synthesis consists of two steps: (1) synthesis of poly(CL-*co*-TMC) (PCT) copolymer via ring opening polymerization of ε-caprolactone and trimethylene carbonate and (2) acrylation of poly(CL-*co*-TCM) copolymer. A series of PCTAc with varying monomer ratios, molecular weights, or acrylate end-capping groups were synthesized. The general procedure for PCTAc 75:25 is described below as an example.

#### 2.2.1 Synthesis of Poly(CL-*co*-TMC) 75:25, 4 kDa

Typically, a 100 mL round-bottom flask was equipped with a magnetic stir bar and charged with TMC (5.00 g, 49 mmol), CL (17.26 g, 147 mmol), TEG (1.16 g, 7.94 mmol), and Sn(II)Oct (57.40 mg, 0.14 mmol). The mixture was heated to 140 °C and stirred for 24 h under a nitrogen atmosphere. The final product was then dissolved in CHCl_3_ and purified by precipitation in cold methanol (∼0 °C). A viscous transparent solid was collected and dried under high vacuum for 48 h (13 g, 60%).

#### 2.2.2 Acrylation of Poly(CL-*co*-TMC) 75:25

There were two methods of acrylation explored for this formulation (75:25, 4 kDa).

In the first method, the printable copolymer (PCTAc) was prepared by reacting the PCT with the LDI-HEA end-capping reagent. Briefly, LDI (3.0 g, 13.33 mmol) was dissolved in anhydrous toluene (5 mL, 47 mmol), and then Sn(II)Oct (60 mg, 0.13 mmol) was added and stirred for 10 min under a stream of dry nitrogen. A solution of HEA (1.28 g, 13.33 mmol) in anhydrous toluene (5 mL, 47 mmol) was added dropwise under vigorous stirring. After the addition of HEA, the reaction mixture was heated to 40 °C for 12 h and kept in the dark. The final product was obtained as a yellow liquid, which was used without further purification. On the other hand, PCT 75:25 (*M*_n_: 3700 g mol^–1^; 10 g, 2.7 mmol) was dissolved in chloroform (30 mL). Then, Sn(II)Oct (23 mg, 0.05 mmol) was added and stirred for 10 min before the LDI-HEA mixture in toluene (4 mL, 5.4 mmol) was added. The mixture was heated to 40 °C for 24 h. The final product was concentrated under reduced pressure and then purified by precipitation in a cold methanol solution (0 °C). A highly viscous yellow solid (8.9 g, 85%) was obtained after drying under vacuum for 48 h.

To compare the effect of the acrylate end-capping group on the shape memory properties, a second PCT acrylate was synthesized by the reaction of PCT 75:25 with acryloyl chloride. Briefly, 5 g (1.3 mmol) of PCT 75:25 4 kDa was dissolved in 30 mL of DCM, under a nitrogen atmosphere, and acrylated by a reaction with a four molar excess of acryloyl chloride (420 μL, 5.2 mmol), which was added dropwise, in the presence of a 2.5 molar excess of TEA (460 μL, 3.25 mmol). The reaction solution was stirred overnight at room temperature. Subsequently, the polymer was precipitated in diethyl ether, filtrated twice, and finally dried under high vacuum (7 g, 75%).

### 2.3 Characterization

Nuclear magnetic resonance (^1^H NMR and ^13^C NMR) spectra of PCT copolymers were recorded on a Bruker ASCEND 700 MHz NMR spectrometer equipped with a TCI cryo probe. Seven milligrams of the sample was taken and dissolved into 700 μL of deuterated chloroform (CDCl_3_). Chemical shifts were studied and correlated to their chemical structure using MestReNova.

Gel permeation chromatography (GPC) was conducted using *N,N*-dimethylformamide (DMF) containing 0.1 wt % LiBr as eluent, and the sample concentration was fixed to 1 g L^–1^. The GPC Shimadzu system was comprised of an autosampler, a Shodex KD-G 4A guard column (4.6 × 10 mm) with 8 μm beads, followed by two Shodex KD-802 (5 μm, 8 × 300 mm) and KD-804 (7 μm, 8 × 300 mm) columns, a refractive index detector, and a photodiode array detector at 50 °C with a flow rate of 1 mL min^–1^. The GPC system was calibrated against linear poly(methyl methacrylate) standards with molecular weights ranging from *M*_p_ = 600 to 265,300 Da. The samples were filtered through poly(tetrafluoroethylene) (PTFE) membranes with a pore size of 0.2 μm prior to injection.

### 2.4 Fabrication of 3D Scaffolds

#### 2.4.1 Preparation of Printing Resins

UV-curable resins were prepared by mixing poly(CL-*co*-TMC) acrylates (70 wt %) with diphenyl(2,4,6-trimethylbenzoyl)phosphine oxide (TPO, 1 wt %) as a photoinitiator and 2-(2-ethoxyethoxy)ethyl acrylate (EOEOEA, 30 wt %) as a reactive diluent, as previously determined.^19,20^ The resins were heated to 50 °C and mixed manually until they were homogeneous.

#### 2.4.2 3D Printing

3D scaffolds (shapes: open box and gripper) were fabricated using a commercially available DLP printer (ARM 10, Roland, Geel, Belgium) with a customized vat and printhead. The scaffolds were printed at temperatures of around 55 °C via a heated vat. The printing parameters used include a layer thickness of 50 μm and a curing time of 3.5 s for all layers. After manufacturing, the scaffolds were washed two times with acetone and ethanol to remove all of the unreacted resin. The scaffolds were then postcured in a UV oven at 365 nm (10 mW cm^–2^) for 5 min from both top and bottom sides.

### 2.5 Shape Memory Behavior

Dynamic scanning calorimetry was performed using a TA Instrument Q 2000 DSC to analyze the thermal properties of photo-cross-linked PCT-based resins. Samples were measured in a range from −80 to 100 °C using heat–cool–heat cycles using a 10 °C/min temperature rate. The melting point was determined at the maximum of the endotherm peak.

#### 2.5.1 Shape Memory Testing at Body Temperature

The printed 3D structures (open box, original shape) for each PCTAc formulation were heated to 40 °C followed by deformation to the desired configuration (closed box, temporary shape) under an external force. The temporary shape was fixed by cooling in a freezer (4 °C). Shape recovery was achieved by submerging the structure in a water bath at 37 °C.

A gripper-shaped structure (based on PCTAc 75:25 4 kDa copolymer) was also printed, and a similar heating–cooling–heating cycle was performed. In this case, shape recovery was achieved by using a heat gun to grab and lift a screw from the bench.

#### 2.5.2 Dynamic Mechanical Testing

The quantitative shape memory tests were carried out using a dynamic mechanical analyzer to determine the storage modulus (*E*′) as a function of temperature. Printed samples (40 mm × 10 mm × 0.5 mm) were subjected to a cantilever beam model at a frequency of 1 Hz. The temperature was increased at a heating rate of 5 °C/min from −20 to 120 °C.

## 3 Results and Discussion

### 3.1 Synthesis and Characterization of the Macromonomer Library

The macromolecular structure, such as molecular weight between cross-links, cross-link functionality, and spatial distribution, influences the overall shape memory performance of PCL-based covalent networks.^24^ In this work, we explored the effect of the macromolecular structure of poly(caprolactone-*co*-trimethylencarbonate) urethane acrylates (PCTAc) on the shape memory properties of DLP-printed scaffolds.

**Figure 1:**
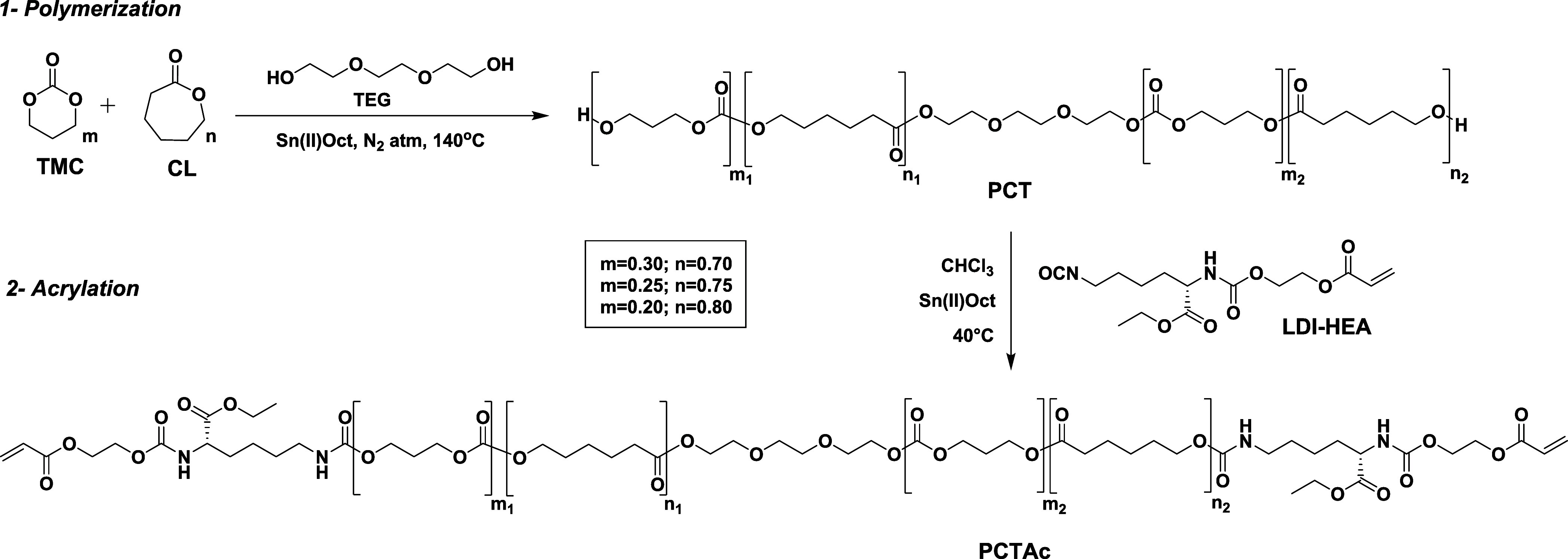
Designing PCT copolymers for DLP printing. Schematic of the synthetic route used to obtain PCTAc macromonomers. Notably, the *m* and *n* copolymer ratios are determined from the feed ratio.

First, we developed a series of poly(caprolactone-*co*-trimethylenecarbonate) (PCT) urethane acrylates (PCTAc) to evaluate the effect of the polymer architecture on the shape memory properties. First, PCT copolymers were synthesized as shown in Figure 1, where caprolactone (CL) and trimethylenecarbonate (TMC) react by bulk ring opening polymerization using triethylene glycol (TEG) as the initiator and Sn(II)Oct as the catalyst. Thus, 5 copolymers were obtained with variation in monomer ratio (80:20, 75:25, and 70:30 CL:TMC, with a *M*_n_ around 4000 g/mol) or molecular weight (4000, 6000, and 12,000 g/mol, with a monomer ratio of 75:25 CL:TMC). The ^1^H NMR spectra of the synthesized PCT copolymers (Figure S1) show the peaks corresponding to the CL and TMC units in the backbone. The peaks were assigned based on previously analyzed structures.^19^ As Figure S1a indicates, we attributed peaks (denoted by a letter followed with an apostrophe) to the formation of dyads, which originated by two units (e.g., CL-CL) next to each other. The monomer composition and molecular weight of all PCT copolymers (Table 1) were determined by NMR analysis. These results show that both monomer composition and molecular weight correspond closely to the target copolymers. The molecular weight dispersities were determined by GPC analysis, showing a range of 1.21–1.99. The dispersities increased with the molecular weight of the copolymer.

**Table 1:** PCT Copolymer Chemical and Thermal Analysis

**PCT** (CL:TMC)	***M*_n_**^a^**(×10**^**3**^g/mol)	**%CL**^a^	**%TMC**^a^	***Đ***^b^	**PCTAc** (CL:TMC)	**acrylation method**	**% acrylation**^a^	***M*_n_**^a^**(×10**^**3**^g/mol)	***Đ***^b^	***T*_m_**^c^**(°C)**
70:30	4.0	62	38	1.40	70:30	LDI-HEA	35	4.2	1.41	23
75:25	3.7	73	27	1.21	75:25	LDI-HEA	51	4.1	1.40	30
					acryloyl	acryloyl chloride	76	3.8	1.45	33
80:20	3.3	76	24	1.23	80:20	LDI-HEA	44	3.6	1.23	32
75:25 6k	5.8	72	28	1.99	75:25 6k	LDI-HEA	63	6.2	1.69	22
75:25 12k	12.3	72	28	1.58	75:25 12k	LDI-HEA	70	12.7	1.66	27

a Determined via ^1^H NMR analysis.

b Determined via GPC in DMF.

c Determined via DSC.

After polymerization, PCT copolymers were modified with acrylate end groups by reaction with a preformed LDI-HEA reagent (Figure 1 (2)) in a 1:1 functional group ratio. Thus, five printable copolymers (PCTAc) were synthesized and characterized by NMR and GPC. The LDI-HEA end-capping reagent was prepared as we reported previously.^19^ The hydroxyl end groups of PCT copolymer react with the isocyanate group of the LDI-HEA end-capping reagent to form photopolymerizable PCT copolymer with acrylate end groups. Furthermore, we also assessed a different acrylation strategy to evaluate the effect of the end groups on the shape memory properties. A PCTAc 75:25 acryloyl was synthesized by acrylation of PCT 75:25 with acryloyl chloride (Scheme S1) in a 1:2 functional group excess. The degree of acrylation, molecular weight, and dispersity were determined by ^1^H NMR (Figure S1) and GPC (Figure S2). The NMR spectra of all PCTAc copolymers (Figure S1) exhibit the peaks around 4.5 ppm corresponding to the vinyl protons (denoted with “u” in Figure S1). The percentages of acrylation were around 53 and 76% for LDI-HEA and acryloyl chloride methods, respectively. GPC analysis showed that copolymers presented dispersities in the range of 1.2–1.7, being the highest values for the high molecular weight PCTAc.

### 3.2 Resin Development and Network Formation

**Figure 2:**
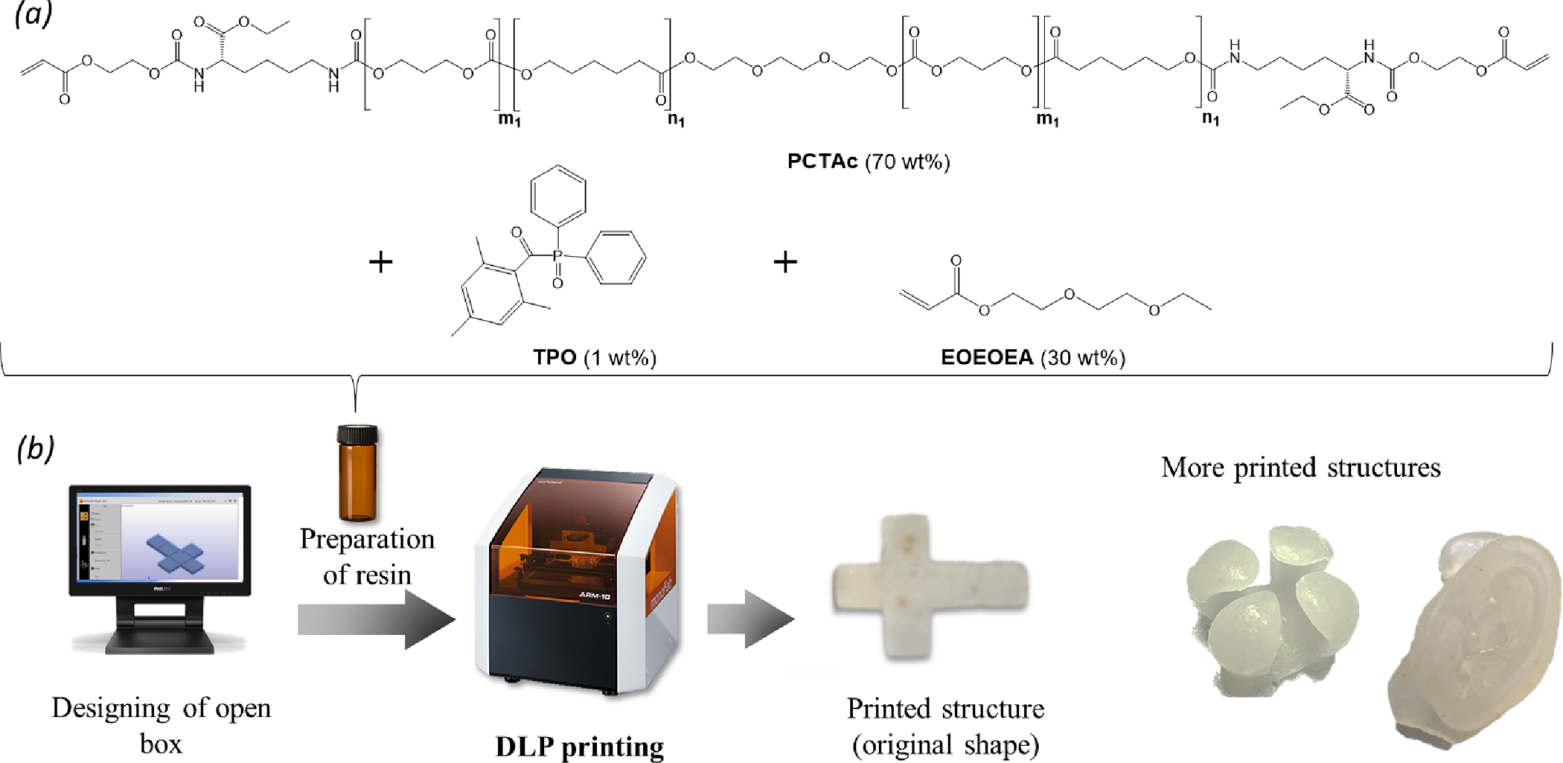
Resin formulation and DLP printing of resins. (a) Preparation of resins. (b) Scheme of DLP printing and images of PCTAc 75:25 printed structures.

PCTAc copolymers are great candidates as biocompatible and bioresorbable DLP resin formulations due to their biodegradability and ability to be formulated into resins with a reactive diluent. The use of a homemade heating stage allowed minimizing the content of the reactive diluent to 30 wt %, which is needed for reducing the resin viscosity and increasing printability. The reactive diluent chosen, 2-(2-ethoxyethoxy)ethyl acrylate (EOEOEA), is a well-established and biocompatible monofunctional acrylate commonly used in dental applications.^29^ Thus, we prepared DLP resins for the new PCTAc library (Figure 2a) following the optimized formulation: 70 wt % of macromonomer, 29 wt % of EOEOEA, and 1 wt % of diphenyl(2,4,6-trimethylbenzoyl)phosphine oxide (TPO, biocompatible photoinitiator).^19,20^ To corroborate if the resin formulation can be used for DLP printing, we prepared a film by casting and photocured it for each formulation. All resins were able to spread uniformly and cross-linked to form freestanding films; for example, the PCTAc 75:25 resin film is shown in Figure S3.

### 3.3 Thermal Characterization of Resins

The shape memory properties of PCTAc require a thermo-mechanical programming process, where the melting temperature triggers the transitions between original and temporary shapes. Therefore, the thermal behavior of the printed structures for all PCTAc formulations was investigated by DSC.

**Figure 3:**
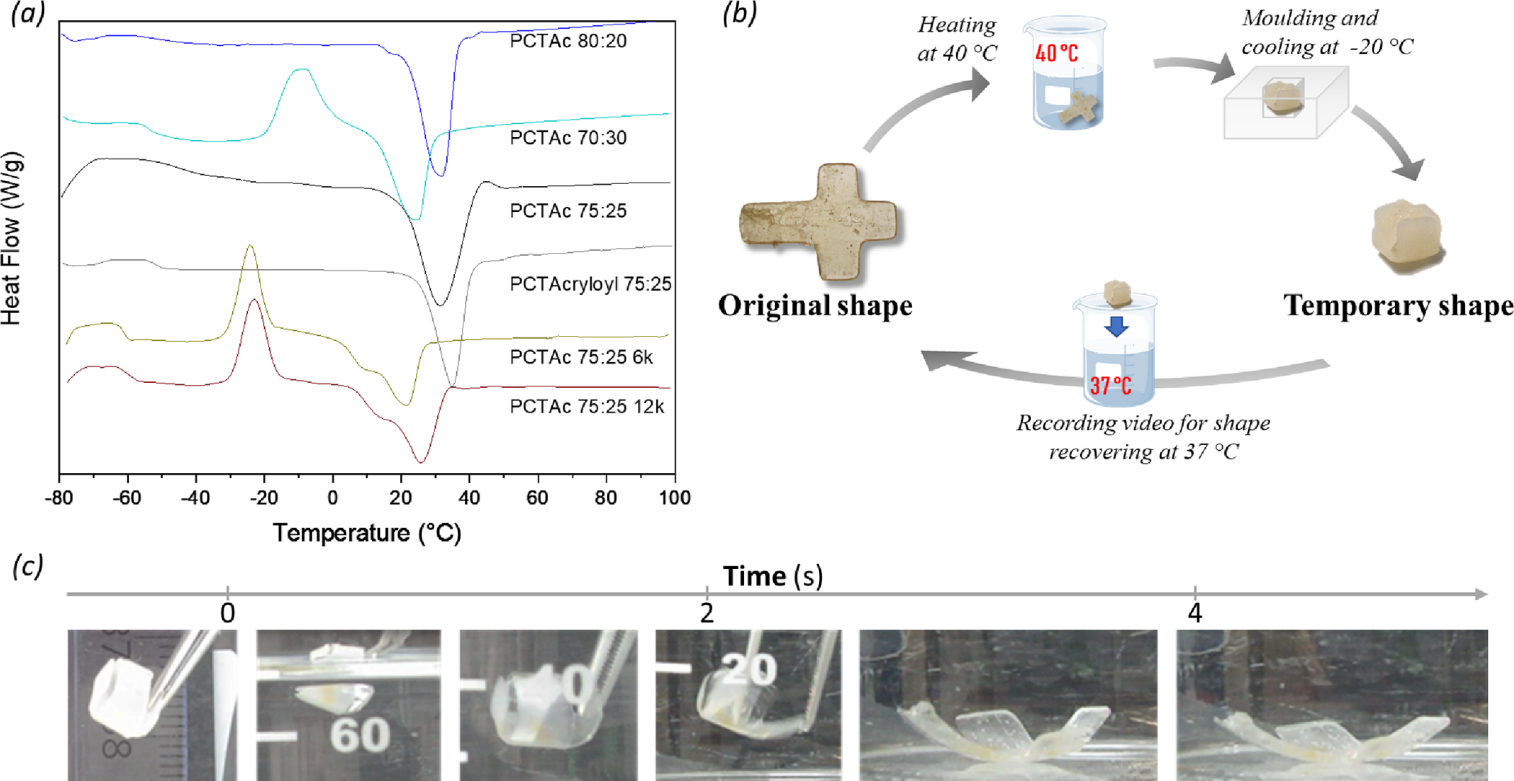
3D-printed scaffolds based on PCT could change their shape at body temperature. (a) DSC graph of printed PCTAc samples. (b) Scheme of the heat–cool–heat cycle. (c) Example of heating cycling for the PCTAc 75:25-based closed box (temporary shape) in a water bath at 37 °C.

Figure 3a shows the DSC heating curves of the PCTAc networks. All samples exhibit melting peaks at around 28 °C (Table 1), which are attributed to the CL crystalline domains within the PCTAc network. The values of *T*_m_ for PCTAc 80:20, 75:25, and 70:30 scaffolds showed that the higher amount of CL within the network increased the transition temperature as expected. The end groups of PCTAc 75:25 also affect the melting point. The extra molecule, LDI-HEA, incorporated at the end of the polymer shifted the melting point to a lower value related to the polymer with only acrylate groups (PCT acryloyl) and broadened the peak. This behavior is probably due to the crystallization disruption. On the other hand, PCTAc 12k and 6k (12 and 6 kg/mol, respectively), with the same monomer ratio (75:25) and end groups (LDI-HEA), showed lower *T*_m_ values compared to the one with a molecular weight around 4 kg/mol. However, the relationship between the melting point and the molecular weight does not show a clear trend. Even though the higher molecular weight can affect the chain movement capacity, the reactive diluent (which is part of the network), the degree of acrylation, and the polymer dispersity (higher values for 12k and 6k, Table 1) may play a role in the crystalline formation. Typically, polymers containing CL units present melting points due to the crystalline domains, where the molecular weight and architecture play a key role in the crystallite formation; however, changes in cross-link density (acrylate functionalization) can also confound this effect. Thus, an increase in melting point can be associated with larger crystalline domains in the network, given that the network has the freedom to form these domains.

All PCTAc samples also showed a crystallization point during either heating or cooling curves. The crystallization temperatures were around −10 °C. Interestingly, the samples with lower melting points showed the crystallization transition in the heating curves, while the other samples showed the crystallization transition during the cooling ramp.

### 3.4 DLP Printing

The merging of high-resolution prototyping technology and shape memory materials makes these 4D-printed scaffolds promising for tissue engineering and biomedical applications, among other fields. Here, we explored DLP technology for fabricating PCTAc-based structures to further study their shape-programmable properties. Open box shapes were first designed (stl file), and then the PCTAc resins were printed using the DLP printer with a homemade heating stage (Figure 2b).^11^ All resins were processable by DLP (Figure S3), and all were fabricated at a printing time of 3.5 s per layer. Only the PCTAc 75:25 12k structure was soft and sticky, making it harder to remove it from the printhead and manipulate.

### 3.5 Modulation of Shape Memory via Polymer Architecture

The melting point observed in all printed PCTAc-based structures was used as a triggering stimulus for shape memory behavior. In this case, a thermo-mechanical programming process is required for the transition from the original shape (printed structure) to the temporary shape. The printed open boxes (original shape) went through a heat–cool–heat cycle, as Figure 3b shows. In the first step, the material is heated above its melting point and deformed to adopt the temporary shape (closed box). Then, the temporary shape is fixed by cooling (kept in the freezer overnight), owing to the crystallization of CL domains. In the last step, the original shape, “memorized” by the material, is recovered by heating at 37 °C (above *T*_m_ for all samples). Thus, we determined the time of shape recovery (box completely open as was printed) at body temperature due to the potential biomedical applications. The effect of monomer ratio, molecular weight, and acrylate end-capping group of PCT copolymers on the shape memory properties of printed scaffolds was studied (Table 2).

**Table 2:** Modulation of Shape Memory via Polymer Architecture

**variable**	**PCTAc**	**shape recovery (s)**
monomer ratio	70:30	2
	75:25	4
	80:20	6
end group	75:25 (LDI-HEA)	4
	75:25 acryloyl	3
molecular weight	75:25 4 kg/mol	4
	75:25 6 kg/mol	2
	75:25 12 kg/mol	35

All printed structures were capable of adopting a temporal shape (close box) after the programming process and recovering their original shape within seconds by putting into a 37 °C water bath. An example of the recovering process is shown in Figure 3c, where the PCTAc 75:25 box takes 4 s to recover its original shape.

The time of shape recovery changed with the copolymer composition, as Table 2 shows, being faster with a higher amount of amorphous monomer (TMC) in the copolymer. As discussed above, the higher amount of TMC decreased the melting point due to crystalline disruption. Consequently, less energy (lower temperature) was needed for the shape transition comparing copolymers with similar molecular weight. Here, we explored the shape recovery at 37 °C due to the relevancy for medical applications. In this series, we can see that a higher difference between *T*_m_ and water bath temperature leads to faster recovery time.

Then, we investigated the effect of end groups on shape memory. The acrylation of PCT 75:25 was carried out by two methods, LDI-HEA and acryloyl chloride, which led to the same copolymer backbone and different end groups. Even though the end groups of the copolymers affect their melting point, the recovering times for them were similar. Probably, the network architecture may play a role in recovering time. PCT acryloyl 75:25 may have a denser cross-linked network than PCTAc 75:25, owing to the higher acrylation percentage, which would drive the sample to recover its original shape faster.

In addition, PCTAc 75:25 copolymers with different molecular weights (4, 6, and 12 kg/mol) were studied. As we see in Table 3, there was not a clear trend in the shape recovery and the molecular weight. PCTAc 75:25 6k showed the lowest melting point, which could be attributed to less crystallinity. Probably, the high dispersity of this copolymer creates less perfect crystallites, and the big difference between *T*_m_ and 37 °C (water bath) makes the structure recovery to its original shape faster. On the other hand, the long polymer chain (higher mobility) of PCTAc 75:25 12k would drive better crystalline domain formation than that of 6k one, resulting in higher *T*_m_. However, this sample showed the slowest shape recovery of all of the copolymers. Taking into account that all resins are made with the same PCT weight percentage, the high molecular weight also creates lower cross-linking density. Therefore, the low amount of cross-links may make PCTAc 75:25 12k slower in shape recovery.

In summary, the recovery time shows that the shape memory properties can be modulated by the network design. In our previous work, we observed that a minimum amount of CL within the copolymer composition is needed for exhibiting the melting point. Here, this transition allows us to develop printed structures with shape memory properties. The network design (copolymer composition and network architecture) affects the crystalline domain formation and, consequently, the shape memory properties. Despite the crystalline domains, the cross-link density may also play a role in the shape recovery.

### 3.6 Mechanical Characterization of Printed Structures

**Figure 4:**
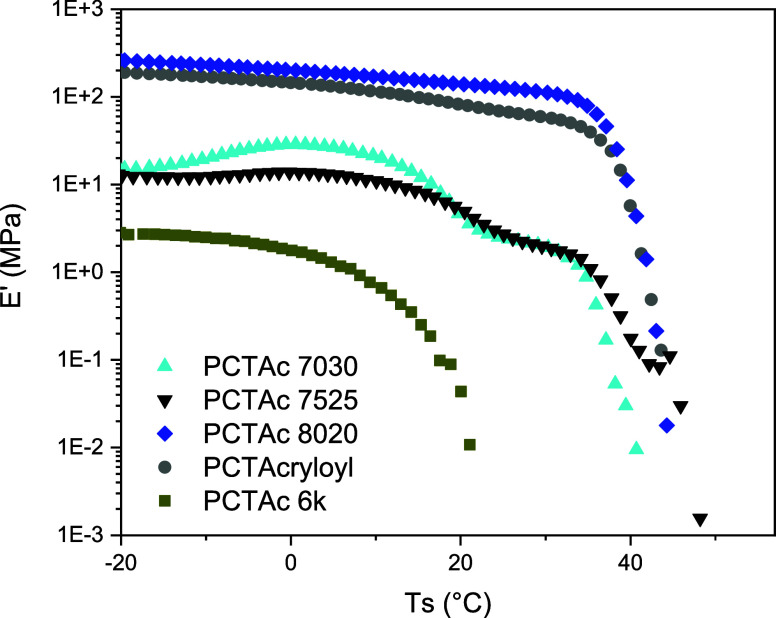
Dynamic mechanical characterization of 3D-printed PCT-based scaffolds. Storage modulus (*E*′) vs temperature curves of PCTAc networks with different polymer architecture.

The thermo-mechanical behavior of printed scaffolds was determined by dynamical mechanical analysis (DMA). As Figure 4 shows, the dynamic mechanical properties of the PCT networks depend on the copolymer architecture. All DMA curves exhibited a sharp decrease in the storage modulus (*E*′) from the rubbery to melt state around 40 °C, except for PCTAc 6k with a state transition around 20 °C.

By analyzing the copolymer composition, *E*′ values of the formulations with higher TMC (PCTAc 70:30 and 75:25) were lower than that of the PCTAc 80:20 network. As discussed above, the larger amount of TMC may disrupt the crystalline domain formation, consequently decreasing the stiffness of the printed material. To analyze the shape recovery transition, we compared *E*′ values at 20 °C (room temperature) and 37 °C (water bath). The drop in *E*′ value with the increase in temperature was attributed to the increasing molecular mobility, which allows for the release of the stored elastic energy as a mechanical restoring force and, thus, material recovery. A higher difference in storage modulus was observed with increasing TMC in the copolymer, which is in good accordance with our recovery time.

The end group of the copolymer has a great impact on the dynamic mechanical behavior. PCTAc 75:25 with acryloyl (small end group) presented higher stiffness in the rubbery region compared to the copolymer obtained by the LDI-HEA method (big end group). The improvement on the mechanical properties could be attributed to two factors: one is a larger crystalline domain for the PCT network with smaller end groups, and the second is the larger cross-linking degree due to the larger amount of acrylate functionalities in the sample (Table 1). However, the shape memory properties were similar for both networks, indicating a delicate interplay between the end groups that will require more work to uncover. Despite the differences in storage moduli in the rubbery plateau, the samples show a similar drop in storage modulus from 20 to 37 °C, which represents the restoring force for shape recovery. To further understand the differences in cross-linking density between samples, swelling of printed resins in a good solvent (chloroform) was performed. As shown in Table S2, we did not observe large differences between the resin samples, suggesting the absence of large differences in effective covalent cross-link network.

The molecular weight also affects the mechanical properties of the printed PCT scaffolds. PCTAc 12k was too soft and sticky at room temperature, making it impossible to test by DMA. As previously mentioned, the high dispersity of the medium molecular weight PCT (6000 kg/mol) may affect the crystalline formation. Consequently, the mechanical performance of PCTAc 6k, as well as its thermal performance, decreased compared with that of PCTAc 4k. The 6k network showed not only low values of *E*′ (low stiffness) at all temperatures but also a fast (and broad) melting transition. Regarding the shape recovery, a change in *E*′ from 20 to 37 °C was slightly higher than that of PCTAc 4k, which is in accordance with the faster observed recovery.

The dynamic mechanical properties, as well as thermal behavior, are affected by the crystalline domains of CL within the network. The DMA curves also showed that the chosen temperatures (20 and 37 °C) for the shape recovery test matter. The drop in *E*′, which greatly influences the shape memory properties, was determined by the copolymer architecture.

### 3.7 Creation of Structures Performing Work

**Figure 5:**
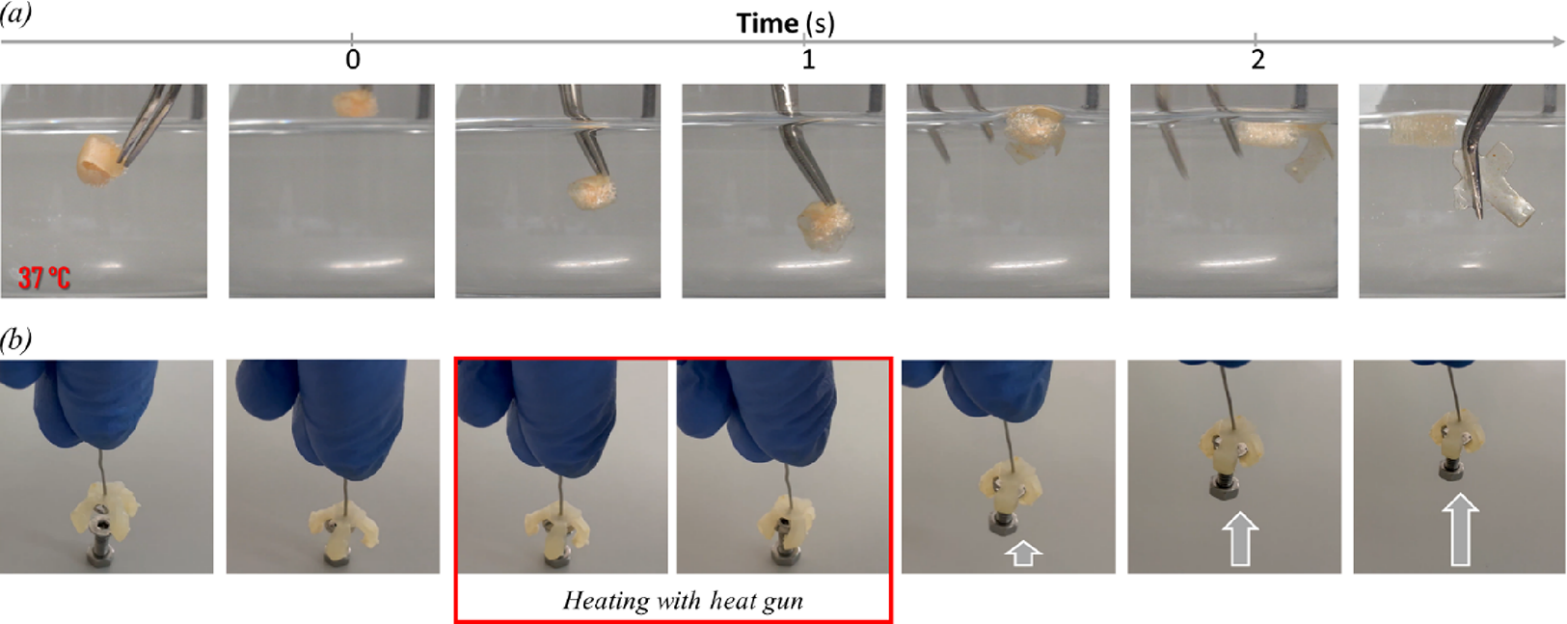
Shape memory-programmable printed PCT-based scaffolds could grab, lift, and release cargo. (a) Closed box (PCTAc 70:30) that released cargo at 37 °C (water bath). (b) Gripper (PCTAc 75:25) closed upon application of heat (via a heat gun) for grabbing and lifting a screw.

Besides the DLP resins developed being great candidates for printing biocompatible and biodegradable complex structures, their shape memory properties make the printed structures promising for drug delivery systems, sensors, minimally invasive procedures, and self-tightening degradable sutures, among others. Here, we were able to show simple delivery of a payload from within the 3D-printed objects, triggered by body temperature (Figure 5a and Video S1). Furthermore, we printed a gripper (Figure 2b) and, after fixing the temporary shape, grabbed a screw (Figure 5b and Video S2) to show the potential in fields such as soft robotics.

## 4 Conclusions

The synthesis of the copolymers has allowed us to explore these DLP-polymerizable resins with programmable shape memory. We found that changes in the copolymer composition (higher PCL content) could lead to slower shape memory recovery or a transition at a higher temperature, while changes in the copolymer molecular weight led to slower shape memory recovery. Interestingly, we found no effect on the end-capping of the polymer on the shape memory effect (LDI-HEA vs acryloyl); however, this did lead to differences in moduli below the shape memory effect temperature. So far, the printed structures have shown quick shape recovery, even more than one cycle, at body temperature. The current results lend these cytocompatible and biodegradable polymers well to further in vitro and in vivo testing. The tunability of these resins can enable slight tailoring to the intended application; for example, biomedical applications may prefer a slow shape memory effect with softer mechanical properties, while soft robotics may favor more rapid shape changes with slightly stiffer materials. We envision the use of these dynamic shape memory polymers as a platform to enable the use of DLP-printed scaffolds for soft and medium-soft tissue regeneration and to explore soft robotics in biomedical applications.

## Supporting Information Available

Shape memory-programmable printed PCT-based (PCTAc 80:20) scaffold in an open box shape that can deliver a foam cargo at body temperature in water (MP4)
Shape memory-programmable printed PCT-based (PCTAc 75:25) scaffold printed in a closed claw shape that can grab and lift a simple bolt upon warming with a heat gun (MP4)
NMR and GPC of all synthesized polymers, images of all printed structures, and speed of shape memory recovery at different temperatures (PDF)
