# Supplementary material for: Digital Light Processing Resins with Programmable Shape Memory for Biomedical Applications
Source: Biomacromolecules. 2024 Jul 29;25(8):4677–85. doi: 10.1021/acs.biomac.3c01276 (PMC11322996; doi:10.1021/acs.biomac.3c01276)
Supplement: Supplementary file 3 — bm3c01276_si_003.pdf [file bm3c01276_si_003.pdf]

DLP resins with programmable shape memory for biomedical applications

Ana A. Aldana, Tobias Kuhnt, Ramiro Marroquin Garcia, Lorenzo Moroni, Matthew B. Baker

Department of Complex Tissue Regeneration, MERLN Institute for Technology Inspired Regenerative Medicine, Maastricht University, P.O. Box 616, 6200 MD Maastricht, The Netherlands.

Supplementary Information

Degree of acrylation

The content of acrylic moieties determined by quantitative NMR. For example, triethylene glycol (TEG) was used as an internal reference to determine the acrylate content (M) through the following formula:[1]

$$M(\%) = \frac{I_u/N_u}{I_{TEG}/N_{TEG}} \times 100$$

Where l_u indicates the integration of the NMR peak between 5.8–6.5ppm, which peaks correspond to the acrylate. N_u is the amount of hydrogens associated with the peak of the acrylate group. While ITEG and NTEG indicate the integration of the peaks between 3.5–3.8 ppm, and the number of hydrogels associated with those peaks, respectively.

Synthesis of Acroyloyl end capped polymer

Scheme S1. ***Synthesis scheme for the direct endcapping of the poly(ester-co-carbonate) to yield the PCT Acryloyl.***

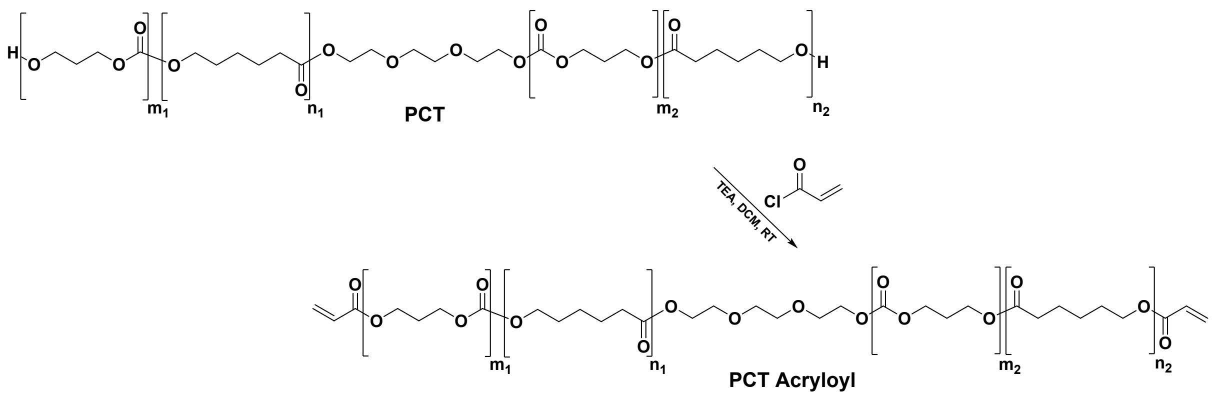

Figure S1. ***^{1}H-NMR of all PCT and PCTAc polymers.***

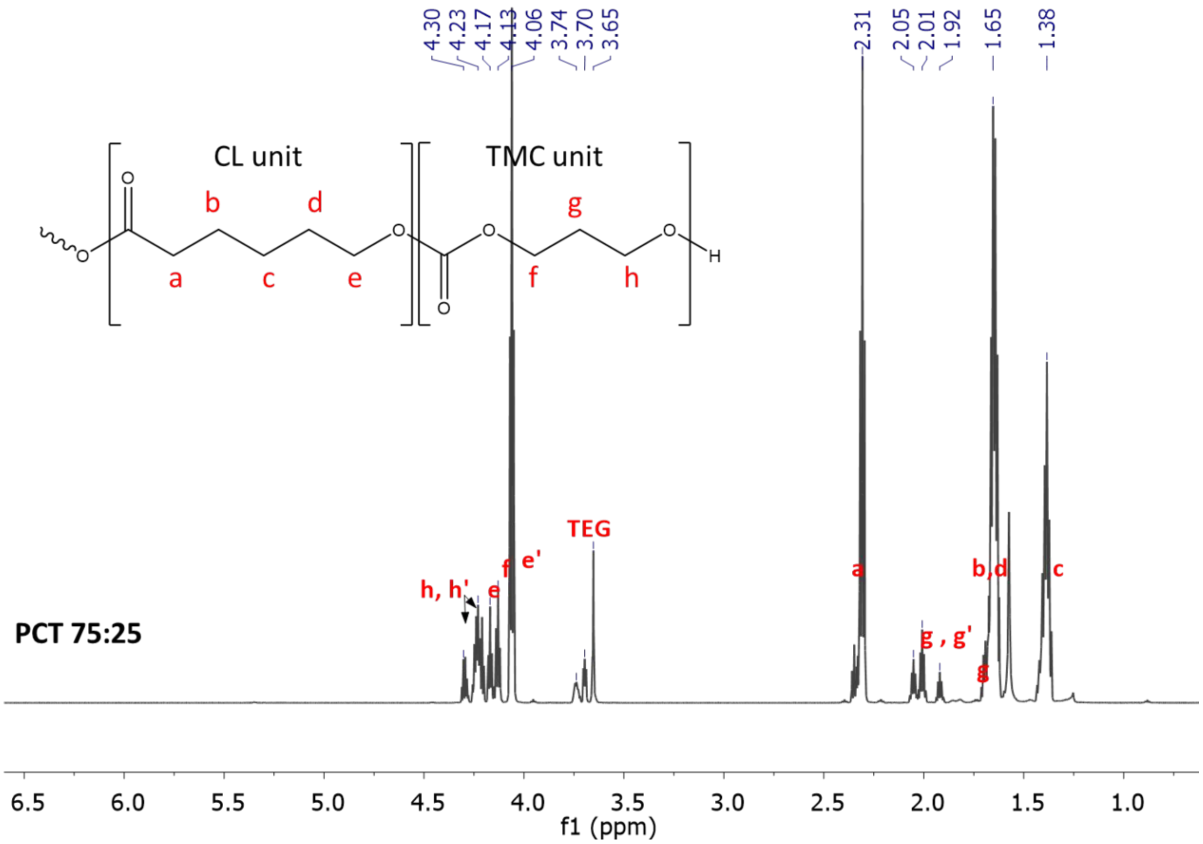

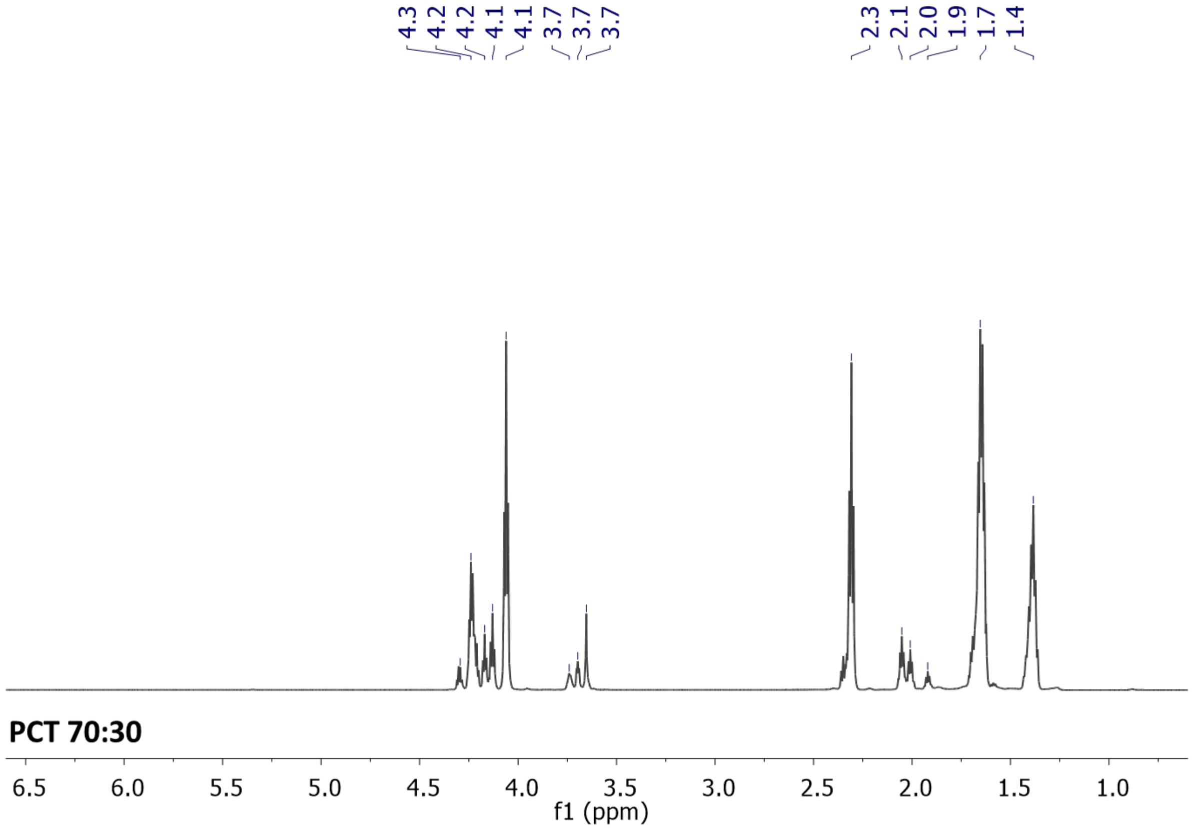

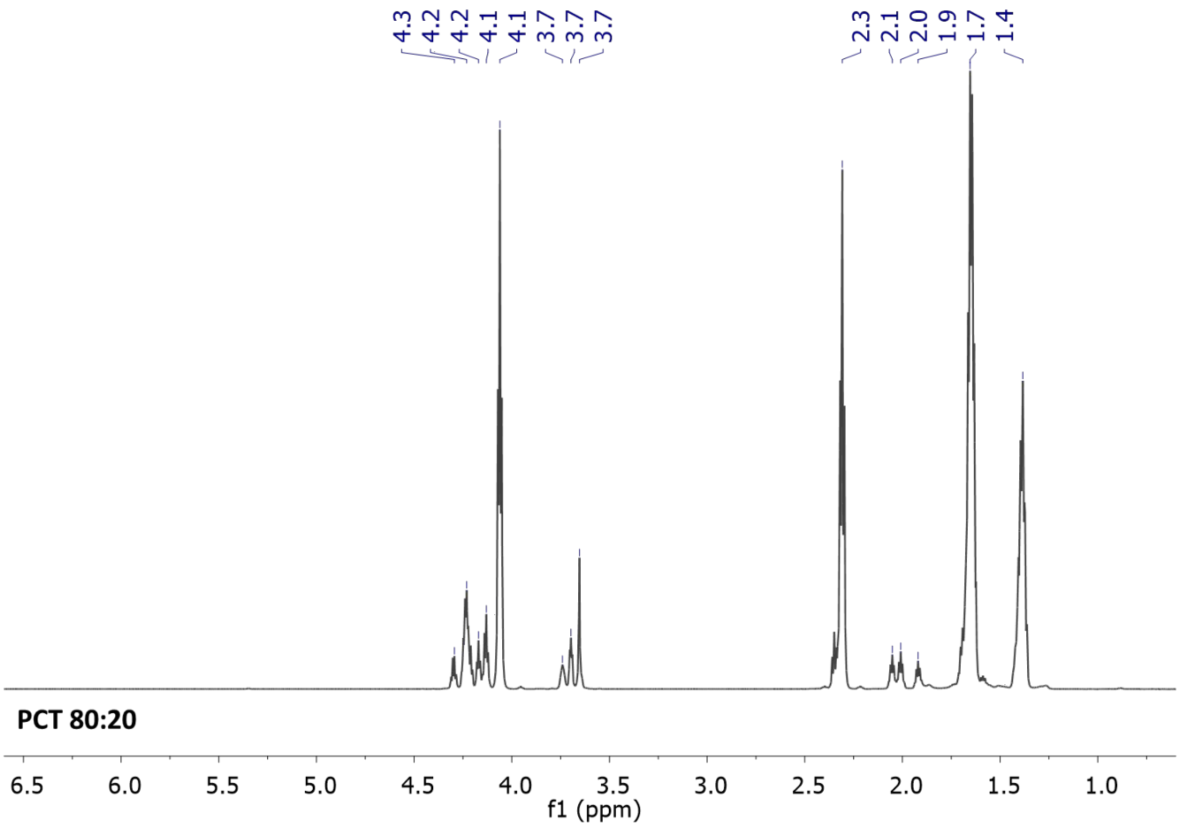
4.3
4.2
4.2
4.1
4.1
3.7
3.7
3.7
2.3
2.1
2.0
1.9
1.7
1.4
PCT 80:20
6.5
6.0
5.5
5.0
4.5
4.0
3.5
3.0
2.5
2.0
1.5
1.0
f1 (ppm)

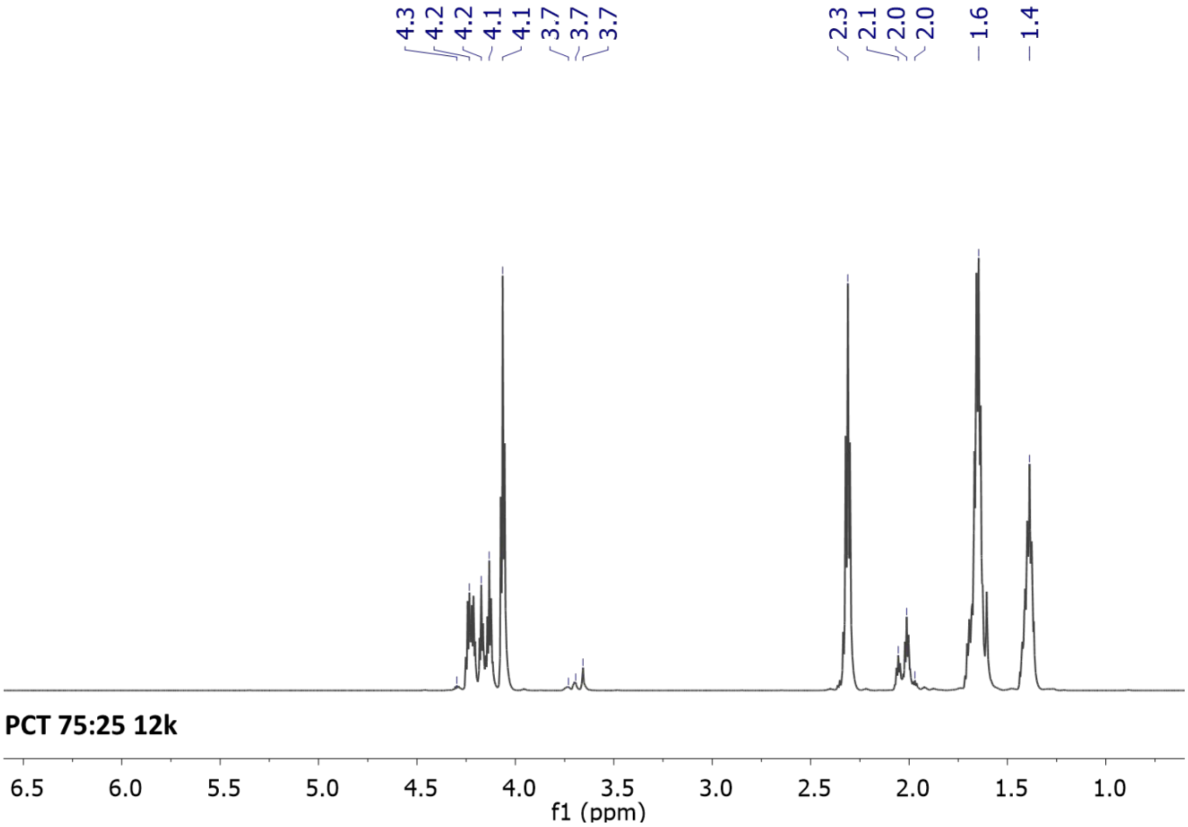
4.3
4.2
4.2
4.1
4.1
3.7
3.7
3.7
2.3
2.1
2.0
2.0
1.6
1.4
PCT 75:25 12k
6.5
6.0
5.5
5.0
4.5
4.0
3.5
3.0
2.5
2.0
1.5
1.0
f1 (ppm)

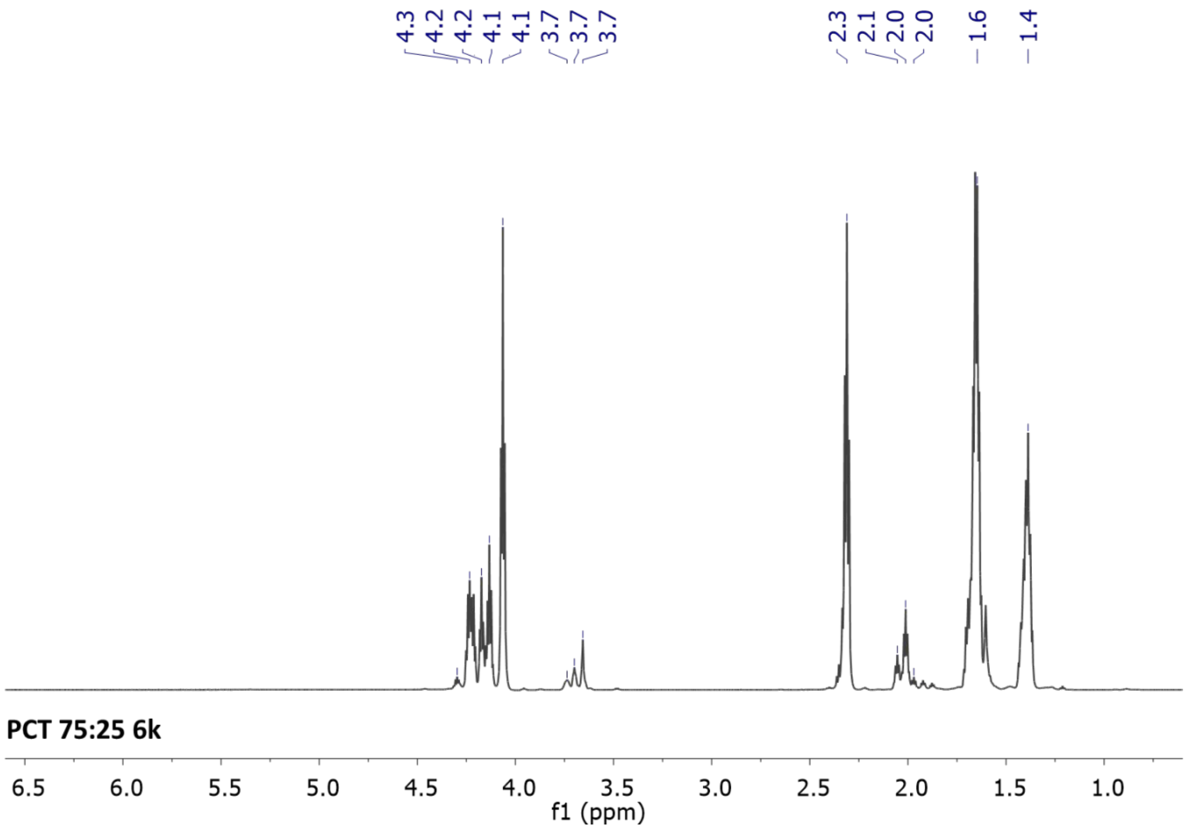

4.3
4.2
4.2
4.1
4.1
3.7
3.7
3.7
2.3
2.1
2.0
2.0
1.6
1.4
PCT 75:25 6k
6.5 6.0 5.5 5.0 4.5 4.0 3.5 3.0 2.5 2.0 1.5 1.0
f1 (ppm)

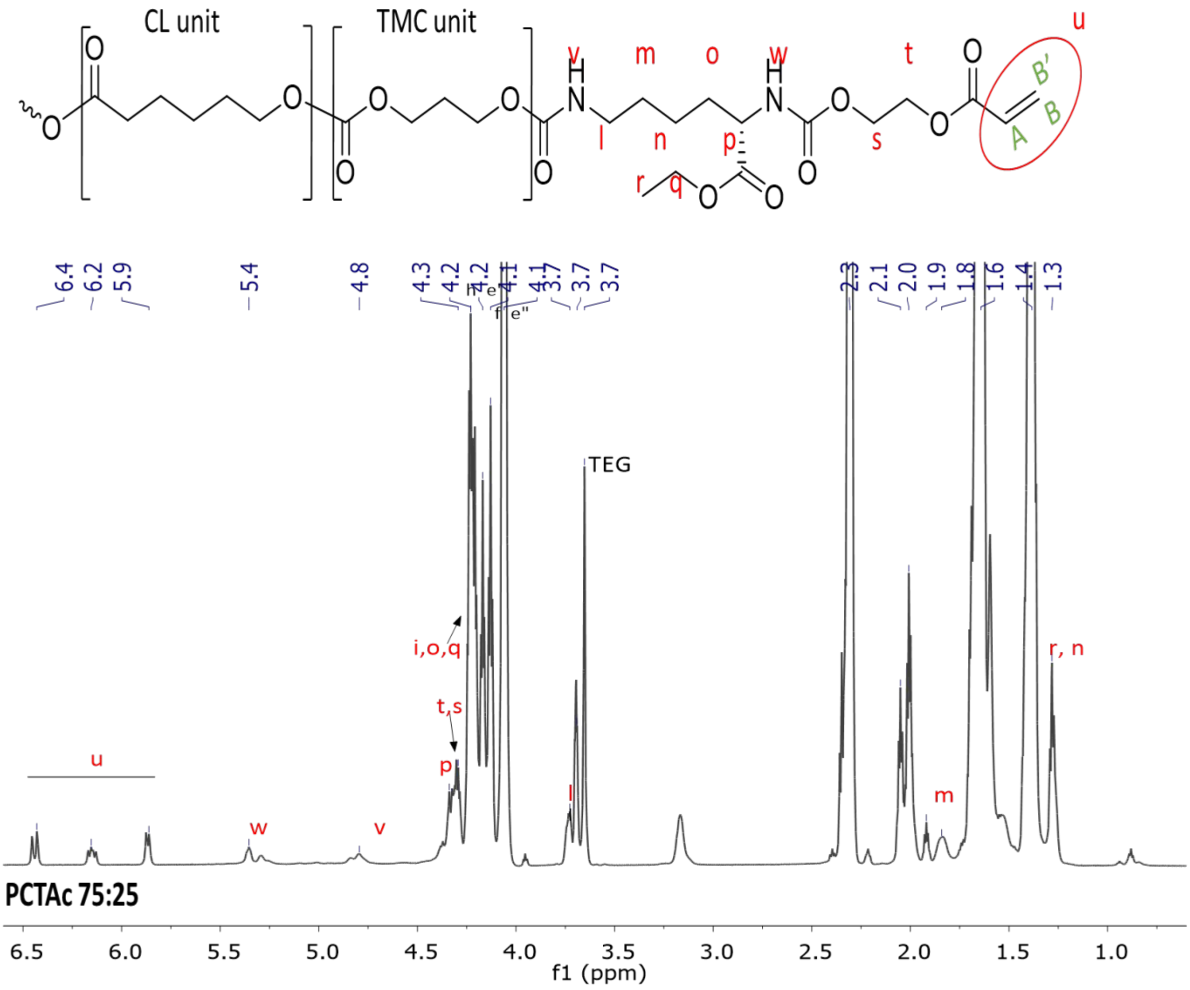

CL unit
TMC unit
u
TEG
i,o,q
t,s
p
l
w
v
m
r, n
PCTAc 75:25
f1 (ppm)

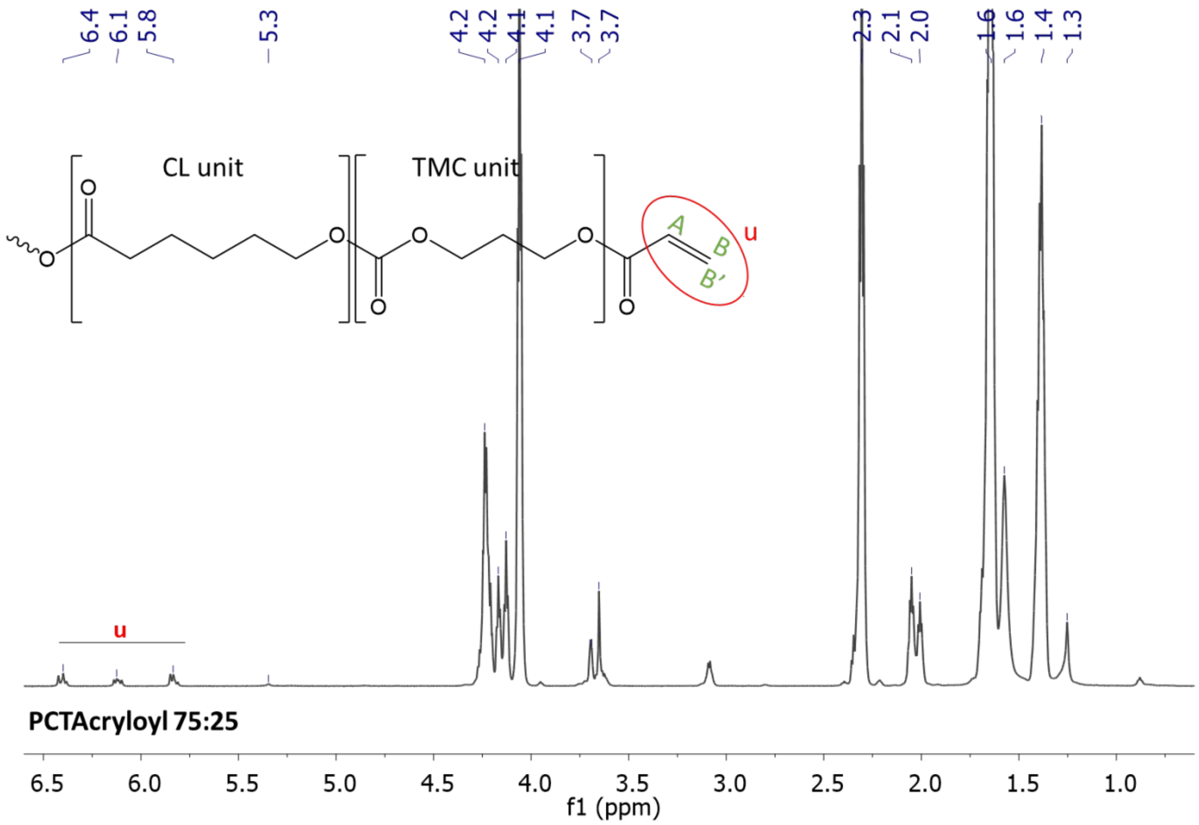
6.4
6.1
5.8
5.3
4.2
4.2
4.1
4.1
3.7
3.7
2.3
2.1
2.0
1.6
1.6
1.4
1.3
CL unit
TMC unit
A
B
B'
u
u
PCTAcryloyl 75:25
6.5 6.0 5.5 5.0 4.5 4.0 3.5 3.0 2.5 2.0 1.5 1.0
f1 (ppm)

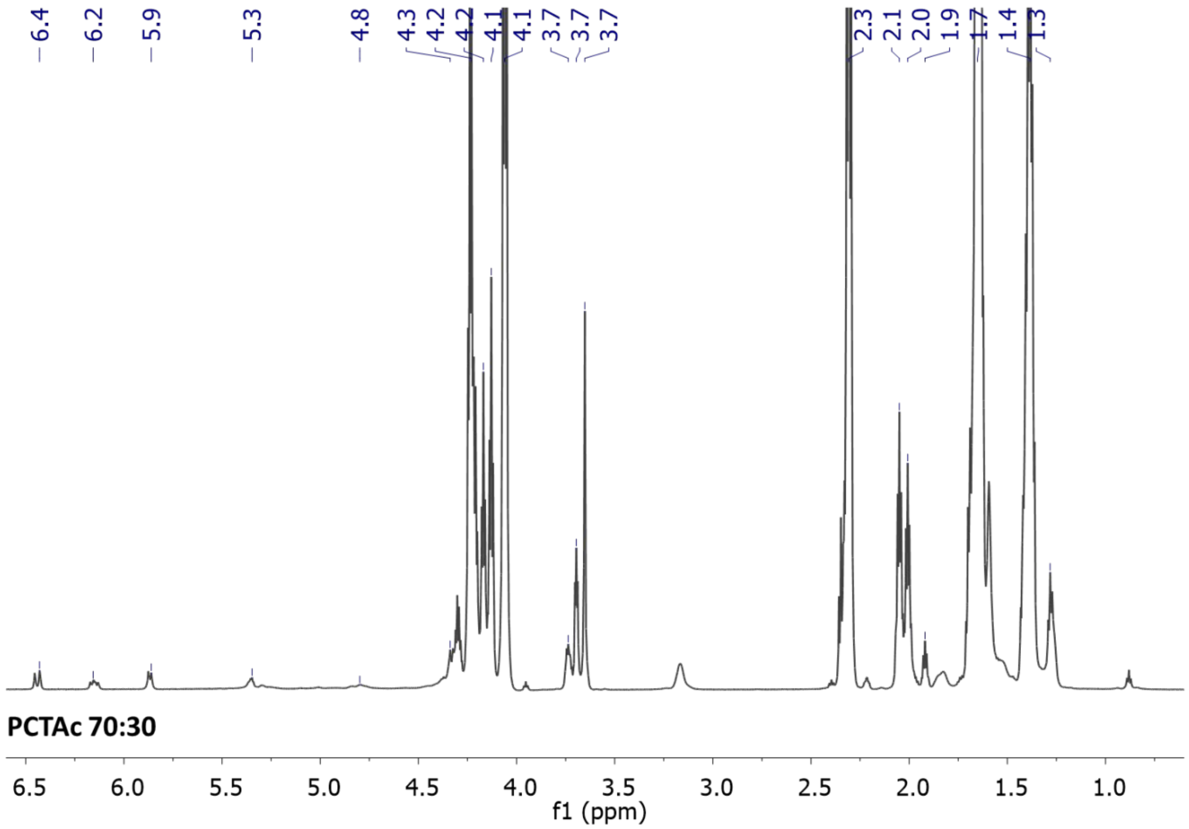
6.4
6.2
5.9
5.3
4.8
4.3
4.2
4.2
4.1
4.1
3.7
3.7
3.7
2.3
2.1
2.0
1.9
1.7
1.4
1.3
PCTAc 70:30
6.5 6.0 5.5 5.0 4.5 4.0 3.5 3.0 2.5 2.0 1.5 1.0
f1 (ppm)

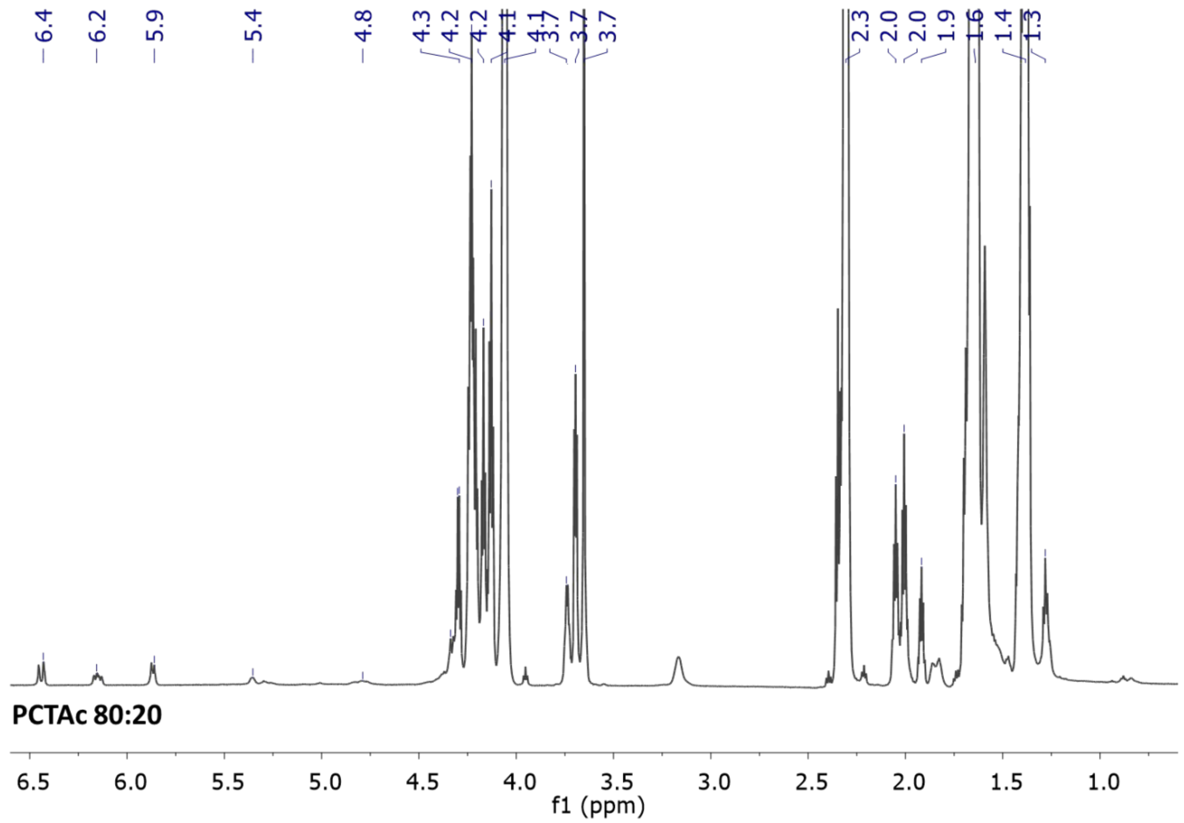
PCTAc 80:20
f1 (ppm)

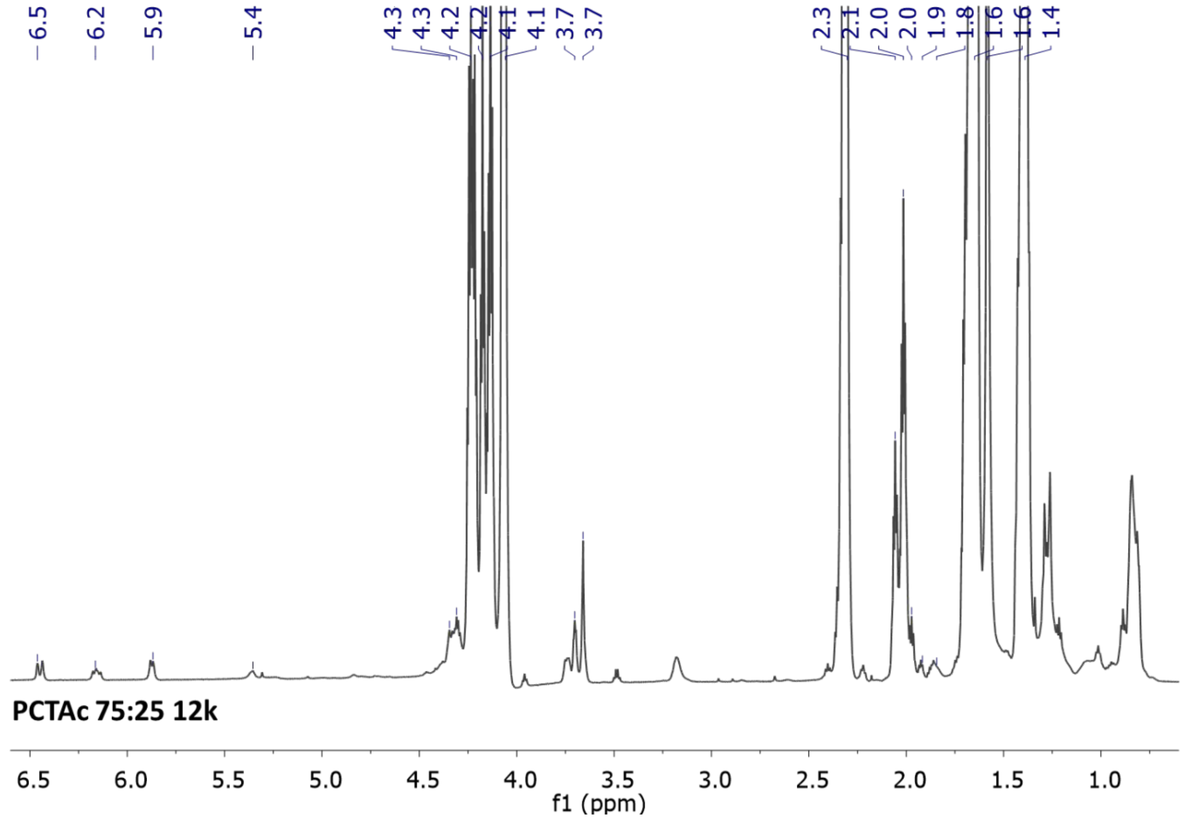
PCTAc 75:25 12k
f1 (ppm)

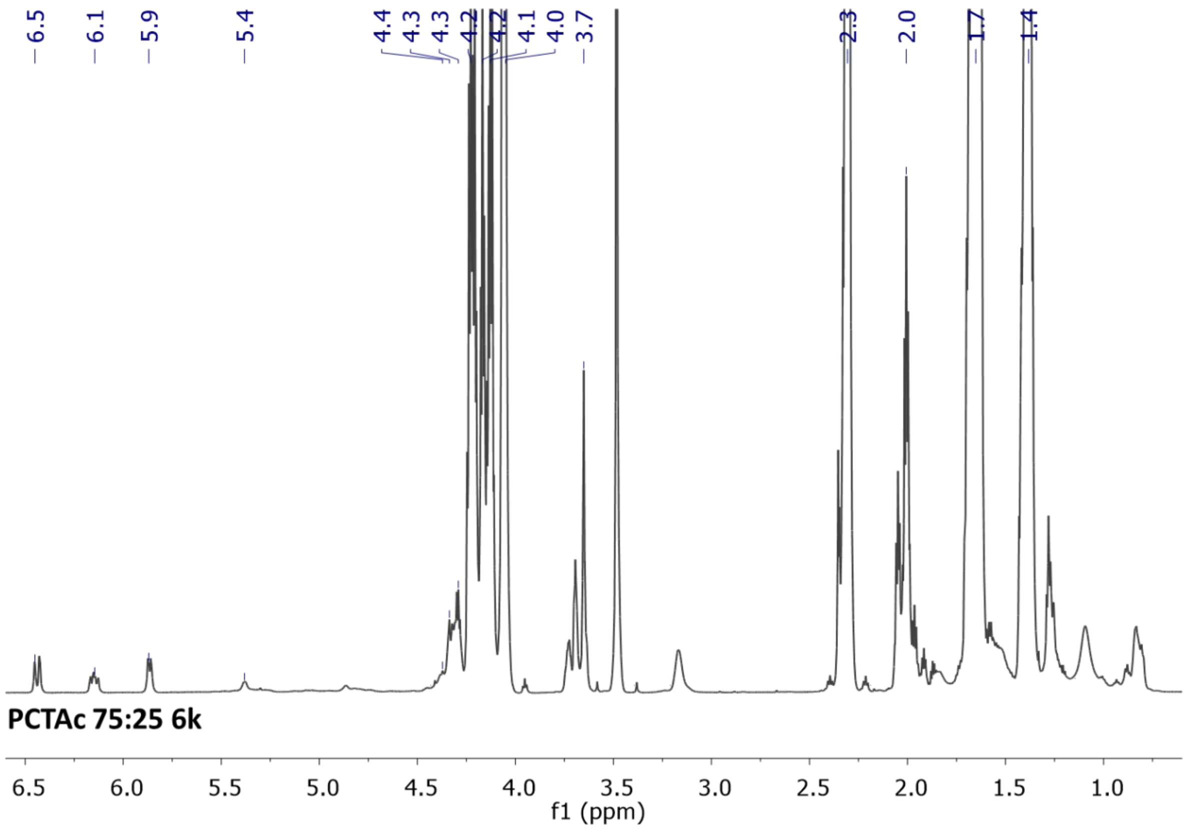

Figure S2. ***GPC of all PCT and PCTAc polymers.***

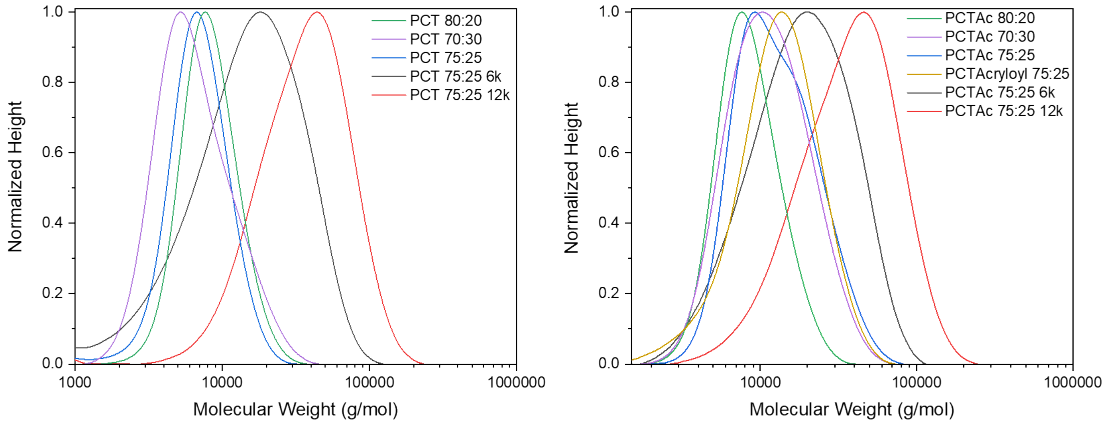

Figure S3. ***Images of all PCTAc printed structures.***

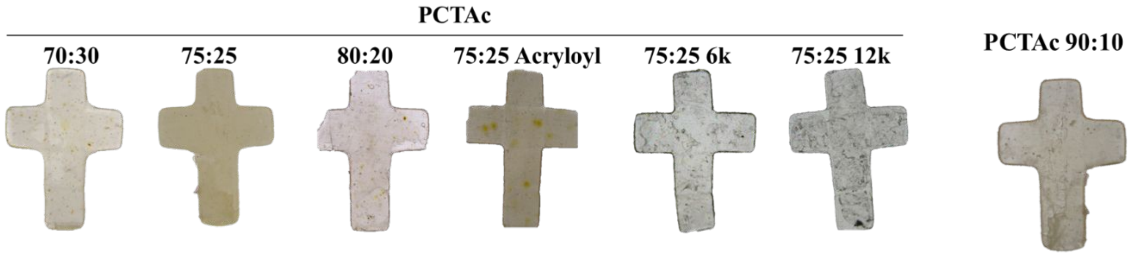

Table S1. ***Shape recovery of PCTAc 90:10 at different temperatures.***

PCTAc	Water bath temperature (°C)	Shape recovery time (s)
90:10	37	N.D.
	40	120
	50	4
70:30	37 (1st cycle)	2
	37 (2nd cycle)	2
	37 (3rd cycle)	2

N.D. means sample did not recover its shape

Table S2. ***Swelling of printed resins in chloroform.***

Sample	Swelling Ratio
PCTAc 70:30	12.3±0.3
PCTAc 75:25	9.5±0.7
PCTAc 80:20	13.6±1.3
PCTAc 75:25	9.5±0.7
PCTAcryloyl 75:25	15.1±1.3
PCTAc 75:25	9.5±0.7
PCTAc 75:25 6k	10.1±2.3
PCTAc 75:25 12k	11.0±1.6

Printed structures (20.0 mg) were submerged in chloroform (5 mL). The samples swelled within 10 min, and they were weighted after 1 hour. The swelling ratio was calculated with the following equation:

$$Swelling\ Ratio = \frac{w_s}{w_d}$$

Where w_d and w_s mean the weight of dried and swollen samples, respectively.

*Videos S1***. *Shape-memory programmable printed PCT-based (PCTAc 80:10) scaffold in an open box shape can deliver a foam cargo at body temperature in water.***

*Videos S2***. *Shape-memory programmable printed PCT-based (PCTAc 75:25) scaffold printed in a closed claw shape, can grab and lift a simple bolt upon warming with a heat gun.***

Figure S4. FT-IR spectra of all PCT printed structures

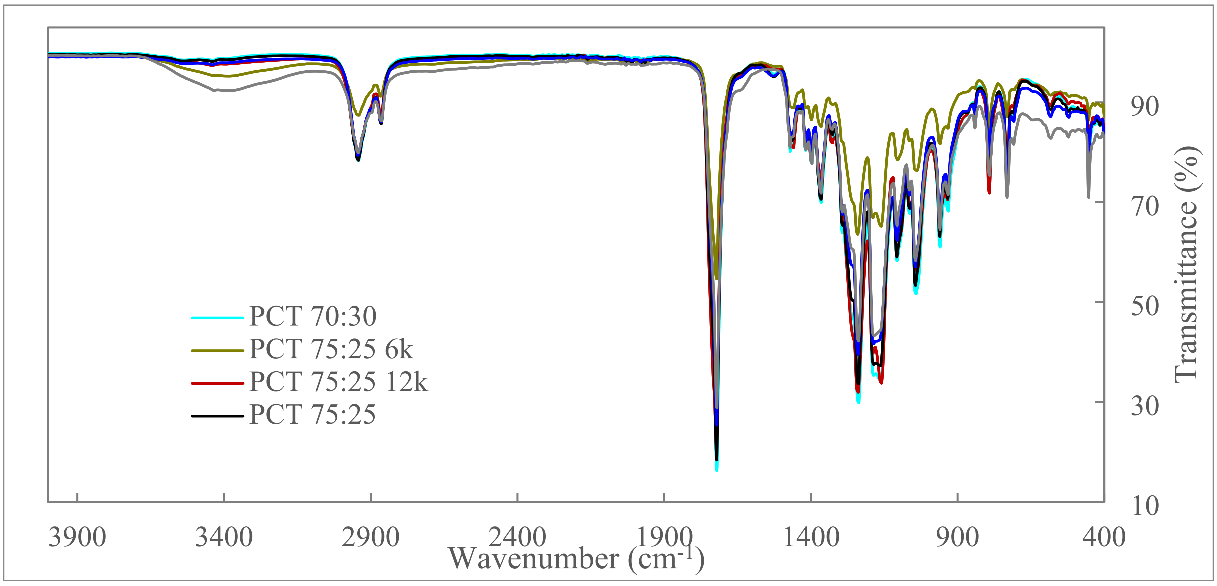

1. Kuhnt, T.; Marroquin Garcia, R.; Camarero-Espinosa, S.; Dias, A.; Ten Cate, A. T.; van Blitterswijk, C. A.; Moroni, L.; Baker, M. B., Poly(caprolactone-co-trimethylenecarbonate) urethane acrylate resins for digital light processing of bioresorbable tissue engineering implants. *Biomater Sci* **2019,** *7* (12), 4984-4989.
